# Comment on Chatwin et al. Waves of Precision: A Practical Guide for Reviewing New Tools to Evaluate Mechanical In-Exsufflation Efficacy in Neuromuscular Disorders. *J. Clin. Med.* 2024, *13*, 2643

**DOI:** 10.3390/jcm13174991

**Published:** 2024-08-23

**Authors:** Jodi Allen, Gemma Clunie, Helen Newman, Claire Slinger

**Affiliations:** 1 The National Hospital for Neurology & Neurosurgery, University College London Hospitals NHS Foundation Trust, London WC1N 3BG, UK; 2 University College London Centre for Medical Imaging, London W1W 7TS, UK; 3 Surgery and Cancer, Imperial College London, London W12 0BZ, UK; g.clunie@imperial.ac.uk; 4 Charing Cross Hospital, Imperial College NHS Healthcare Trust, London W6 8RF, UK; 5 Barnet Hospital, Royal Free London NHS Foundation Trust, London EN5 3DJ, UK; helen.newman3@nhs.net; 6 University College London Division of Surgery and Interventional Science, London WC1E 6BT, UK; 7 Royal Preston Hospital, Lancashire Teaching Hospitals NHS Trust, Preston PR2 9HT, UK; claire.slinger@lthtr.nhs.uk

We read with interest the paper published by Chatwin et al. [1], Waves of Precision: A Practical Guide for Reviewing New Tools to Evaluate Mechanical In-Exsufflation Efficacy in Neuromuscular Disorders in the April 2024 edition of the Journal of Clinical Medicine. The ways in which the respiratory (physio)therapy profession is embracing new tools to refine the efficacy of mechanical in-exsufflation (MI-E) are both innovative and fascinating, and we would like to congratulate the authors for establishing such an informative review. We do, however, have a couple of issues to query. As members of a speech and language therapy (SLT) group with a special interest and expertise in upper airway assessment using ultrasound and other adjuvant tools, we believe that this review is missing reference to contemporary literature specific to two key upper airway assessment methods: (1) cervical auscultation and (2) ultrasound. We write this letter in the interest of drawing the reader’s attention to this work. In the absence of empirical evidence, expert opinion plays an important role in clinical decision-making and the development of practice guidelines [2]. However, where evidence does exist, a balanced critique of current literature is necessary. This includes recognising the limitations of emerging assessment tools to ensure that readers are well-informed and that risks of misapplication are mitigated [2,3,4]. We hope our contribution will encourage appropriate use of cervical auscultation and ultrasound in clinical practice and facilitate a well-balanced understanding of their potential and, more importantly, constraints as tools to evaluate the upper airway.

## 1. The Challenges of Acoustic and/or Sonographic Assessment of the Upper Airway

The upper airway, from supra- to sub-glottis, is a complex biomechanical system, reflecting its central roles in breathing, swallowing, and voice. Due to its small, cartilaginous, air-filled structure, there is a high risk of both acoustic and/or sonographic artefact interfering with the signal, either from air or adjacent anatomical structures. It is vital to understand how this artefact limits the application of the tools used to assess the upper airway, so we can mitigate against or account for them within the assessment. Below, we describe some of the limitations of cervical auscultation and ultrasound in the assessment of the upper airway. A brief description of both approaches is provided in Table 1.

## 2. Auscultation to Detect Upper Airway and Laryngeal Closure during MI-E

Chatwin et al. state that throat auscultation can be used to gain information on laryngeal airflow and synchronisation of glottic closure to MI-E cycles, akin to the use of cervical auscultation (CA) to evaluate swallowing. Whilst we acknowledge that correlations between the acoustic signal of high-resolution CA and laryngeal vestibular closure have been described in the literature [5,6], these highly advanced technologies remain in their infancy. Their accuracy in detecting physiological events remains in debate, with no robust evidence directly linking vocal fold kinematics with an easily interpretable acoustic signal [7]. A lack of audible airflow might, for example, be due to central swallow apnoea, breathing pattern changes, loss of signal from the transducer, or other biomechanical events such as false vocal fold adduction, arytenoid movement, or epiglottic deflection.

CA has an increasingly demonstrable role as a valid and reliable tool to detect aspiration; however, this is only the case when acoustic signals are obtained and interpreted by trained clinicians using a strict protocol [8]. For any instrumental assessment of vocal fold movement, use of a clear protocol is key, as is knowledge and understanding of technicalities such as transducer selection, placement, and signal analysis [7]. An assessment protocol should include tasks that elicit both vocal fold abduction and adduction, neither of which can be adequately judged from repetitions of the vowel sound /i:/. Minimal suggested parameters of assessment might include repeated sniff plus phonation, breath hold, Valsalva, pitch range, and speech tasks if judgement is to be made about vocal fold mobility by clinicians trained in discriminating acoustic qualities of healthy and impaired vocal function [9,10].

In summary, more research is required to fully understand the application of CA as a diagnostic tool for upper airway dysfunction. CA may be able to indicate a change in airflow but cannot yet confirm the causal mechanism. As such, studies akin to those already underway that utilise gold-standard endoscopy as a reference test for emerging novel assessments such as waveform analysis [11] and ultrasound [12] are certainly indicated. Until these studies have been conducted, attempts to draw conclusions about vocal fold function from CA may lead to erroneous assumptions about the anatomical and functional status of the vocal folds.

## 3. Ultrasound to Evaluate the Upper Airway and MI-E Efficacy

The authors correctly state that there are no published studies on the use of ultrasound to evaluate airway responses to MI-E. There are, however, several studies where ultrasound has been used to study the upper airway both at rest and in function, including recent reviews evaluating its role as a laryngeal and swallowing assessment tool [13,14,15].

Chatwin et al. suggest that ultrasound assessment of the glottis is best conducted via the thyroid cartilage. In our experience, the membranous windows of the thyrohyoid and cricothyroid are preferable anatomical locations to view the vocal folds in adults over the age of 35. Beyond this age, calcification of the thyroid cartilage becomes increasingly common, particularly in males, which prevents US wave penetration through to deeper structures [16,17]. US via the thyroid cartilage would therefore be challenging for many patients living with MND/ALS, where symptoms of sporadic disease most commonly develop between 58 and 63 years, with a greater preponderance in men [18].

Based on our training in sonographic assessment of head and neck anatomy and peer rating work as an SLT ultrasound group, we would like to propose that the labels provided in Figure 16A more likely reflect the false vocal cords. This is because the false cords are fatty in nature and appear hyperechoic compared with the true vocal folds, which are composed of muscle [19]. We therefore believe that the vocal folds labelled in Figure 16A are the false folds. Due to image attenuation, the lower third of the figure is unclear, thus the arytenoid cartilage(s), which would usually present as paired hyperechoic structures, cannot be discerned. The smooth and convex shape of the image labelled thyroid cartilage in Figure 16B increases the possibility that this structure represents cricoid cartilage, particularly as it lacks the thyroid notch. The area labelled vocal folds is most likely to be reverberation artefact given its anatomical alignment with the air-mucosal interface often perceived on the anterior wall of the trachea [20]. We would also argue that discrimination of the arytenoid cartilages (AC) in Figure 16C,D is not clear enough to confidently report adduction of the ACs in this view, given the absence of any clear anatomical landmarking. Noel et al. provide clearly landmarked images of the true and false folds and arytenoid cartilages in the transverse view should the reader wish to familiarise themselves with sonographic laryngeal anatomy [21]. Beale et al. also offer a comprehensive overview of laryngeal anatomy in both transverse and parasagittal views [22].

Bony and heavily calcified structures offer high levels of acoustic impedance. As such, ultrasound waves immediately reflect into the probe, casting an ‘acoustic shadow’ over underlying structures. These shadows play a helpful role in anatomical landmarking. In Figure 17A,B, the hypoechoic shadow to the right of each image is the familiar landmark cast by the hyoid bone. Unfortunately, the lack of acoustic contrast in the soft tissue structures labelled in Figure 17A–D (specifically the tongue and palate) means any movement in these structures during MI-E will be difficult to validate and replicate. In Figure 17E,F, the hypoechoic epiglottis is clearly defined and landmarked inferiorly by a bright white air-mucosal interface. However, the description of epiglottic movement during insufflation is not reflected in the imaging; in both images, the epiglottis is held superiorly, with no evidence of movement sufficient to obturate airflow. It is likely that inferior movement of the epiglottis would result in loss of signal and, therefore, view.

Whilst the ability of ultrasound to identify head and neck anatomy has strengthened in recent years with the emergence of new software and hardware, localisation of anatomy remains a significant challenge. For patients with minimal calcification, normal tonicity, no dysphagia, and absence of secretions (for example, a thyroid surgery population), the literature reports good visualisation of structures via ultrasound [23]. This is not yet true for a neurogenic patient population, and diagnostic test accuracy studies are lacking. We hope we have helped to highlight the challenges in landmarking and validating ultrasound investigations of the oropharynx and upper airway. We also recognise the opportunities this work provides to develop collaborative projects between members of the multidisciplinary team who have an interest in the upper airway. We would welcome further interdisciplinary work to improve the validity and reliability of ultrasound applications in airway assessment and management to enable future translation into practice.

## 4. Summary and Recommendations for Future Practice

The expert opinion piece by Chatwin et al. aims to describe the multiple areas of potential assessment of MI-E for clinicians, particularly respiratory (physio)therapists. This includes CA and US of the upper airway. Much of the primary research and acquisition of practice-based evidence for these techniques have been completed by SLTs, including a study identifying key research priorities and directions for this area in US [24]. The importance of reliably understanding sonographic signals for landmark identification or acoustic signals to comment on vocal fold function should not be overlooked before using US or CA either clinically or as part of complex interventions. Collaboration between professions such as physiotherapists and SLTs will help to achieve this goal, as demonstrated by existing work exploring the application of upper airway assessment tools against a criterion reference standard [11,12]. Understanding patient experience is also vitally important to the selection and implementation of such tools in the future.

It is an exciting time in the development of assessments for the upper airway, and we would like to see more collaboration between upper and lower airway specialist professionals. The engagement of SLT in the assessment of upper airway function when managing patients predisposed to both upper and lower airway malfunction will result in much improved outcomes for patients [25,26].

## Figures and Tables

**Table 1:** Description of cervical auscultation and ultrasound in the context of upper airway assessment.

Approach	Application
Cervical auscultation	Use of a stethoscope, microphone, or electronic sensor to enable clinicians to listen and interpret breath–swallow coordination and swallowing sounds.
Ultrasound	Use of high frequency sound waves to evaluate dynamic movements of the upper airway. Sound waves are generated from a transducer, sent into the body and reflected to form an image.
